# Supplementary material for: Comprehensive Annotation and Functional Exploration of MicroRNAs in Lettuce
Source: Front Plant Sci. 2021 Dec 24;12:781836. doi: 10.3389/fpls.2021.781836 (PMC8739914; doi:10.3389/fpls.2021.781836)
Supplement: Supplementary file 3 [file Data_Sheet_9.ZIP › GSM2230751.plot/Lsa-miRN518_Lsat_1_v5_gn_4_173121.1_2117_TPlot.pdf]

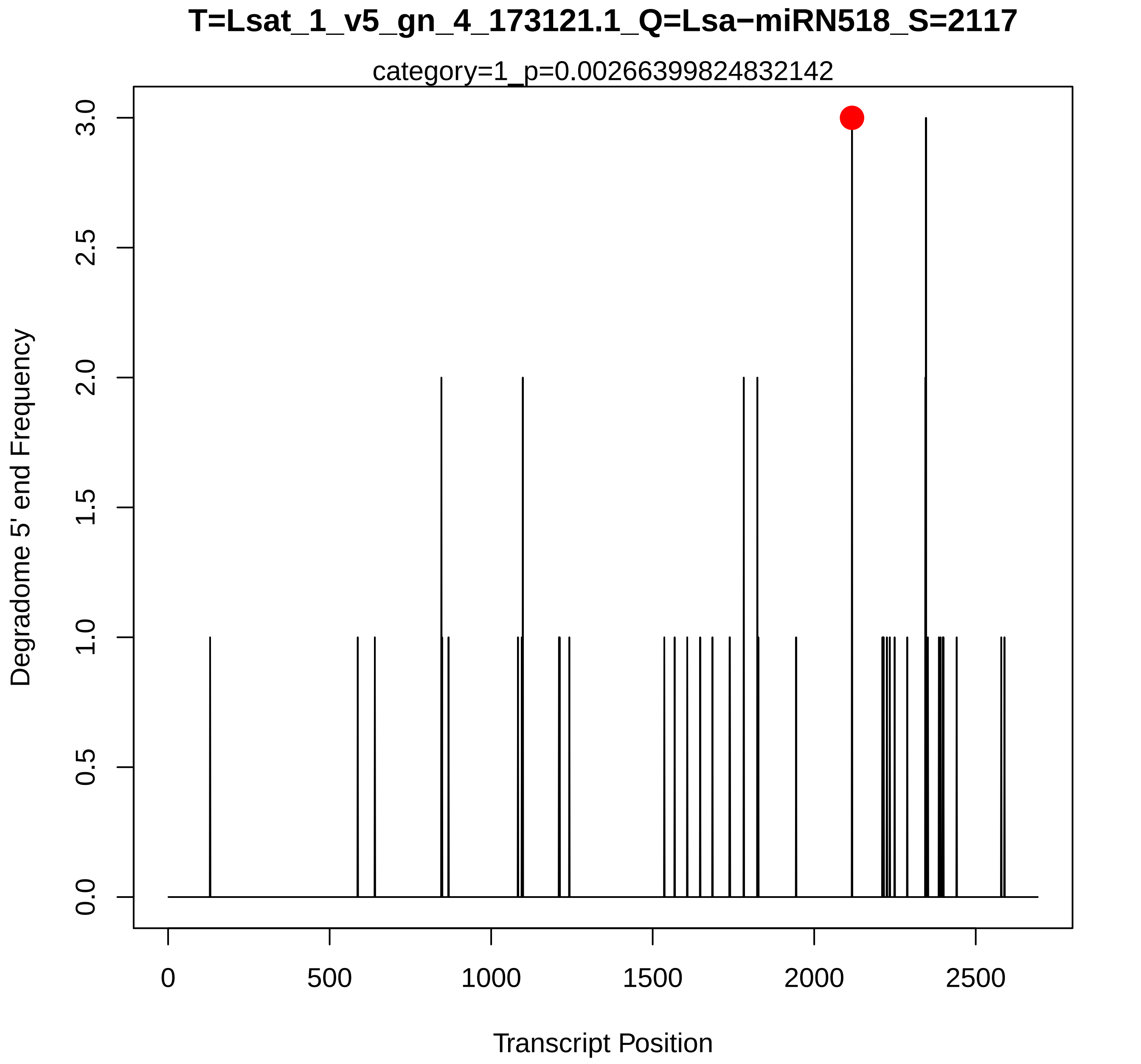

T=Lsat_1_v5_gn_4_173121.1_Q=Lsa−miRN518_S=2117
category=1_p=0.00266399824832142
Degradome 5' end Frequency
3.0
2.5
2.0
1.5
1.0
0.5
0.0
0
500
1000
1500
2000
2500
Transcript Position
